# Derivatization of Hyaluronan to Target Neuroblastoma and Neuroglioma Expressing CD44

**DOI:** 10.3390/pharmaceutics16060836

**Published:** 2024-06-20

**Authors:** Giau Van Vo, Kummara Madhusudana Rao, Ildoo Chung, Chang-Sik Ha, Seong Soo A. An, Yang H. Yun

**Affiliations:** 1 Department of Bionano Technology, Gachon Bionano Research Institute, Gachon University, 1342 Seongnam-daero, Sujeong-gu, Seongnam-si 13120, Gyeonggi-do, Republic of Korea; gvo@sbpdiscovery.org; 2 Degenerative Diseases Program, Sanford Burnham Prebys Medical Discovery Institute, San Diego, CA 92037, USA; 3 School of Chemical Engineering, Yeungnam University, Gyeongsan-si 38541, Gyeongbuk-do, Republic of Korea; msraochem@yu.ac.kr; 4 Department of Polymer Science and Engineering, Pusan National University, Busan 46241, Gyeongsangnam-do, Republic of Korea; idchung@pusan.ac.kr (I.C.); csha@pusan.ac.kr (C.-S.H.); 5 Department of Biomedical Engineering, The University of Akron, Akron, OH 44325-0302, USA

**Keywords:** hyaluronan, resveratrol, brain tumor, CD44

## Abstract

Therapeutics for actively targeting over-expressed receptors are of great interest because the majority of diseased tissues originate from normal cells and do not possess a unique receptor from which they can be differentiated. One such receptor is CD44, which has been shown to be highly overexpressed in many breast cancers and other types of cancer cells. While CD44 has been documented to express low levels in normal adult neurons, astrocytes, and microglia, this receptor may be overexpressed by neuroblastoma and neuroglioma. If differential expression exists between normal and cancerous cells, hyaluronan (HA) could be a useful carrier that targets carcinomas. Thus, HA was conjugated with resveratrol (HA-R), and its efficacy was tested on cortical–neuroblastoma hybrid, neuroblastoma, and neuroglioma cells. Confocal and flow cytometry showed these cells express CD44 and are able to bind and uptake HA-R. The toxicity of HA-R correlated well with CD44 expression in this study. Therefore, conjugating resveratrol and other chemotherapeutics to HA could minimize the side effects for normal cells within the brain and nervous system and could be a viable strategy for developing targeted therapies.

## 1. Introduction

HA is an anionic, non-sulfated glycosaminoglycan that is abundantly expressed by both the embryonic and adult central nervous system (CNS) [1,2,3,4]. Various tissues in the brain express HA and have been shown to surround myelinated fibers [5], neuronal cell bodies [6,7], neurites [8], and neural stem/progenitor cells [4]. HA is especially localized on the dendrites, filopodia, axons, and synapses of mature neurons [8]. Although relatively diffused in white matter [9], HA forms the backbone of perineuronal nets, which are a specialized lattice-like structure and are an integral component of the extracellular matrix of the cerebral cortex and other grey matter regions [8]. Perineuronal nets have also been shown to stabilize synaptic function, maintaining the neuronal microenvironment and controlling synaptic plasticity [4,8]. Although subject to controversy, evidence suggests neurons synthesize HA throughout their maturational stages, but its function beyond the role associated with perineuronal nets, to date, is unclear [8]. In pathological conditions, the over-expression of HA is linked to tumor development [10]. Lung, breast, colon, kidney, prostate carcinomas, astrocytoma, and non-Hodgkin’s lymphoma are also associated with increased amounts of HA in both the epithelial and the intertumoral regions of tumors [11]. HA is known to contribute to cancer motility and migration of various neuronal tumors [12] by increasing the aqueous space within the interstitial tissue, forming larger conduits, and allowing easier passage of cancer cells from tissues to systemic or lymphatic circulations.

The primary ligand for HA was the CD44 receptor [13]. CD44 and other receptors, such as RHAMM, have the ability to both bind and uptake HA [14]. The overexpression of CD44 was linked to tumorigenic mechanisms, such as cell proliferation, metastasis, and migration, and to traits characterized in cancer stem cells such as epithelial–mesenchymal transition, self-renewal, and resistance to chemotherapeutics [15]. The regulation associated with CD44 signaling was paradoxical because HA receptors were observed to have a dual role in which both the over-expression and down-regulation of CD44 could lead certain types of tumors to grow and advance [14]. Thus, conflicting results were associated with CD44 expression and negative clinicopathological outcomes, such as higher tumor histological grades, advanced tumor stages, and poorer survival rates [15]. These results could be partially explained by alternative splicing. Tumors expressing CD44v (variant) isoforms generally seem to be more aggressive than ones expressing CD44s (standard) [16,17,18]. Furthermore, CD44v appeared to confer the metastatic properties to tumors expressing variant receptors [16,17,18]. In contrast, non-metastatic tumors lacking CD44v expression are associated with better patient prognosis but can be highly invasive once they become metastatic [19].

Despite the alternative splicing of CD44, targeting this receptor is an attractive strategy for developing receptor-mediated therapeutics due to the ability of a single HA chain to simultaneously bind multiple CD44 receptors and to signal numerous cancer-associated mechanisms. Even if various CD44 isoforms are expressed on the cell’s surface, HA will still bind to these isoforms of CD44, but its affinity can vary [19]. To induce HA toxicity, anticancer drugs such as resveratrol can covalently bond to its backbone. The potential to develop resveratrol into a chemotherapeutic was first reported in 1997 by Jang et al. [20], when it was shown to inhibit dimethylbenzanthracene (DMBA), inducing pre-neoplastic lesions in an orthotopic mammary mouse model. These results reduced the incidence and growth rate of induced papilloma in mouse skin [20]. The potential intracellular mechanism of this therapeutic outcome included upregulation of p53 and Fas as well as the inhibition of AP-1 (Activating Protein-1), MAPKs (mitogen-activated protein kinases), NF-κB (nuclear factor kappa-light-chain-enhancer of activated B cells), tubulin polymerization, and adhesion molecules [21]. Multiple mechanisms of resveratrol may be useful for treating a wide range of cancers, including neuronal tumors, and could be advantageous if coupled with an appropriate drug delivery system. However, resveratrol alone is a relatively weak drug in comparison to FDA-approved chemotherapeutics [22,23].

The poor water solubility of resveratrol and high metabolic clearance rate [24] were the main reasons for its poor bioavailability. The reported half-life of resveratrol ranged from 8–14 min [24,25]. Thus, resveratrol must be conjugated to water-soluble drug delivery devices to increase its efficiency and shield it from enzymatic activities before reaching cellular targets [22,23]. Due to the poor solubility of resveratrol in an aqueous environment, several delivery methods, such as zinc–pectin-chitosan composite particles [26], acryloyl dimethyl taurate/vinylpyrrolidone hydrogels [24], calcium–pectinate beads [25], solid lipid nanoparticles [27], and synthetic polymer conjugation [22,23] have been investigated. Encapsulation of resveratrol has been shown to enhance its transport to the cell’s cytoplasm by the appropriate carriers, but resveratrol without modification lacks solubility after it has been released. The derivatization of resveratrol with synthetic polymers that are also hydrophilic can offset resveratrol’s solubility, but these conjugates lack receptor targeting unless further conjugated with a targeting moiety.

The conjugation of resveratrol to HA is an attractive approach to increasing this drug’s bioavailability because HA’s backbone contains functional groups for derivatization. HA also extensively forms hydrogen bonds with water, and tethering small amounts of resveratrol to a high-molecular-weight HA could force resveratrol to become soluble in aqueous solutions. Finally, many carcinomas overexpress receptors such as CD44 and RHAMM that bind and uptake HA. HA derivatized to various chemotherapeutics is currently used as a targeting molecule to treat lung [22,28], breast [23,29], colon [29], ovarian [29], and liver carcinomas [22]. Exposing cancer cells to HA derivatized with a mild therapeutic drug could provide a unique therapy for treating neuronal tumors. Therefore, the expression of CD44 was investigated in various neuronal tumors to confirm receptor-mediated attachment and to quantify the efficacy of HA-R.

## 2. Materials and Methods

### 2.1. Synthesis of HA-R

Hyaluronan (HA, 1.35 × 10^6^ g/mole, Sigma-Aldrich, St. Louis, MO, USA) was conjugated to resveratrol using N-(3-Dimethylaminopropyl)-N′-ethylcarbodiimide hydrochloride (EDC, Sigma-Aldrich) at an approximate mass ratio of 5:4:20. HA was dissolved in distilled and deionized water (diH_2_O) at 5 mg/mL at 4 °C. Resveratrol (TCI America) was dissolved in absolute ethanol at 8.33 mg/mL. EDC was dissolved in ethanol at 32.90 mg/mL. Briefly, 6 mL of resveratrol and 6 mL of EDC were added to a round-bottom flask. While stirring at 300 RPM, 8 mL HA was added dropwise, and the reaction proceeded for 20 h in dark conditions. The production of the reaction was then dialyzed (ThermoFisher, Waltham, MA, USA, 10,000 MW cutoff) for 8 h in a 50–50 ethanol/water solution with an exchange of fresh solution after 4 h. The external solution was changed to *diH_2_O*. The next morning, the external solution was exchanged again, and the product was dialyzed for another 8 h. The dialysis was performed in the absence of light to reduce the transformation of resveratrol from a trans- to a cis-conformation.

Hyaluronan (HA) was conjugated to fluorescein isothiocyanate (HA-FITC) using the above procedure, except resveratrol was replaced with FITC. After dialysis, all conjugated products were shell-frozen, lyophilized, and stored at −20 °C until usage. For experimentation, HA-R and HA-FITC were reconstituted at 5 mg/mL in *diH_2_O*. The conjugated products were then diluted to their final concentration using the appropriate aqueous solvents.

### 2.2. Characterization of HA-R

HA-R was characterized using thin-layer chromatography (TLC), NMR, and UV–VIS spectrophotometry. DiH_2_O was added to resveratrol, HA, a mixture of HA and resveratrol (no conjugation), or HA-R. Two microliters of each solution were placed on the TLC paper and placed into a mobile phase of 1:9 acetonitrile–ethanol solution (Sigma-Aldrich, St. Louis, MO, USA). After 30 min, the TLC paper was exposed to UV light. To expose the migration of the HA component for each sample, the TLC paper was exposed to heat to oxidize HA.

HA-R was dissolved in deuterium oxide at a concentration of 5 mg/mL, and ^1^H nuclear NMR spectroscopy was carried out at 500 MHz (Unity-Inova 500). The degree of substitution of resveratrol was determined using a UV−Vis spectrophotometer (SpectraMax M2, Molecular Devices, San Jose, CA, USA). A standard curve of the free drug was generated at 320 nm and compared to the absorbance from 0.03 µg/mL of HA-R to determine the percentage of drug substitution (*w*/*w*) for the conjugate.

### 2.3. Cell Culture

Primary fibroblast (N9) was a gift from Dr. Bum Joon Park, a Professor at Pusan National University. N9 was cultured in Minimum Essential Medium Eagle (MEM, ThermoFisher), 10% fetal calf serum, and 1% antibiotic–antimycotic. N9 was then converted to Dulbecco’s Modified Eagle Medium (DMEM, ThermoFisher). Cortical–neuroblastoma hybrid (A1G11), brain neuroglioma (H4), neuroblastoma (SH-SY5Y (ATCC^®^ CRL-2266)), and human embryonic kidney (HEK293) were purchased from ATCC, cultured in DMEM, 10% fetal calf serum, and 1% antibiotic–antimycotic.

### 2.4. Cellular Uptake

Cell lines and fibroblasts were seeded using the appropriate cell culture medium to a final density of 1 × 10^4^ cells/well in 6-well plates with the presence of sterile German glass coverslips (ThermoFisher). The next day, cells were treated with HA-FITC at 50 µM/mL for 24 h. HA-FITC cells were washed with phosphate-buffered saline (PBS, pH 7.4) three times and fixed with 4% paraformaldehyde diluted in PBS for 10 min. The fixed cells were stained with 4’6-diamidino-2-phenylindole (DAPI, 1 μg/mL) and rhodamine phalloidin (ThermoFisher) according to the manufacturer’s instructions. After washing three times with PBS, the cells in each slide were mounted with Fluromount g (Southern Biotech, Homewood, AL, USA) and sealed for confocal microscopy (Nikon TE2000-E Eclipse C1si). Cells were imaged at an excitation wavelength of 405 nm for DAPI, 488 nm for FITC, and 561 nm for rhodamine.

### 2.5. CD44 Expression

Cells were imaged with confocal microscopy by seeding them onto glass coverslips as previously described [23]. Before the application of the primary antibody, cells were fixed with 1% Formalin for 10 min. Afterward, the fixed cells were rinsed twice with PBS containing 0.05% Tween 20 and permeabilized with 0.2% Triton X for five minutes. The cells were rinsed twice in PBS containing 0.05% Tween 20 and blocked with 1% newborn calf serum for 10 min with the two exchanges of the blocking solution after incubation. Monoclonal anti-CD44 antibody (ThermoFisher) was applied at a 1:200 dilution and incubated overnight. The next day, the cells were washed three times with 0 PBS containing 0.05% Tween 20 and blocked again. A solution of goat anti-mouse antibody conjugated with FITC, rhodamine phalloidin, and DAPI was then applied at dilutions of 1:200, 1:200, and 1 μg/mL, respectively, and incubated for one hour. After washing three times with PBS, the cells in each slide were mounted with Fluromount g (Southern Biotech) and sealed. As controls, cells were prepared as previously stated, but these samples lacked the anti-CD44 antibody; however, the secondary antibody, rhodamine phalloidin, and DAPI were applied.

Flow cytometry was performed using suspended cells. After culturing each cell line and fibroblast, cells for each type were trypsinized, normalized to 1 × 10^7^ cells, suspended, and fixed in 1% formalin. The cells were washed by centrifugation at 3000 rpm and resuspension in PBS. The permeabilization, blocking, and application of the antibodies, rhodamine phalloidin, and DAPI were identical to the confocal microscopy procedure, except the supernatant for each step was separated from the cells by centrifugation since the staining procedure was applied to the cell suspension rather than attached cells. The final resuspension before flow cytometry analysis was in PBS. The primary antibody was applied to the control cells, but the staining solution lacked the secondary antibodies and only contained rhodamine phalloidin and DAPI. The volume for all buffers was the same for each cell type.

### 2.6. Lysosomal Tracking

Cells were seeded onto glass coverslips and exposed to HA-FITC as previously described. The next day cells were stained with LysoTracker™ Deep Red (ThermoFisher) at a 50 nM final concentration in PBS for 15 min. Following labeling, cells were washed and fixed with 4% formaldehyde in PBS. The cells in each slide were mounted and sealed as previously described. Confocal images were obtained with an excitation wavelength of 405 nm for DAPI, 488 nm for HA-FITC, and 633 nm for the LysoTracker.

### 2.7. Cytotoxicity Study

A1G11, H4, SH-SY5Y, HEK293, and N9 were cultured in a 96-well plate with a cell density of 2 × 10^4^ cells per well. The next day, standard curves were produced by replacing the cell culture medium with a medium containing either HA-R (based on the amount of resveratrol), HA, or resveratrol. The final drug concentrations were 100, 50.0, 25.0, 12.5, 6.25, 3.5, 1.75, and 0.00 μM (control). The HA concentrations were matched to the HA-R concentrations. After incubation for 72 h, wells were washed with PBS three times, and cell culture medium (100 μL) was exchanged. Afterward, 100 μL of CellTiter-Glo^®^ Luminescent reagent (Promega, Madison, WI, USA) was added to measure the cell viability. After gently shaking for 10 min, the luminescence signal was measured using a microplate reader (Perkin Elmer, Victor X5, Waltham, MA, USA). The percentage of cell viability was calculated based on luminescence absorbance changes by comparing absorbance values between the test and the control samples. The experiments were repeated three times for statistical analysis.

### 2.8. Data Analysis

The histograms from the flow cytometry analysis of CD44 were analyzed using Photoshop. The individual images were imported into this software (version 23.4.1) where the white area under the histogram was filled. Using the select tool, only the area under the histogram was selected, which excluded all other graph components such as the x- and y-axis. The number of pixels within the histogram was determined using the color range function. The number of pixels for each cell type was then normalized by the largest value obtained.

Regression analysis was applied to the standard curves for HA-R to determine the linearity and the amount of resveratrol conjugated to HA. The cell toxicity studies were performed in triplicate for each experimental condition and each cell type. After the raw data were obtained, the cells without treatment (only containing cell culture media) were averaged and used to normalize the OD results for the corresponding cells treated with HA-R, HA, or resveratrol. The resveratrol samples were supplemented with 1% ethanol to dissolve this drug. Four-parameter logistic (4PL) regression analysis was applied to calculate the IC_50_ values. In addition, each group was tested for normality using the Shapiro–Wilk test. If the dataset was normal, an ANOVA with Tukey’s post hoc test was applied. Data were determined to be significantly different if the *p*-value was less than 0.05.

## 3. Results and Discussion

### 3.1. Characterization of HA-R

**Figure 1:**
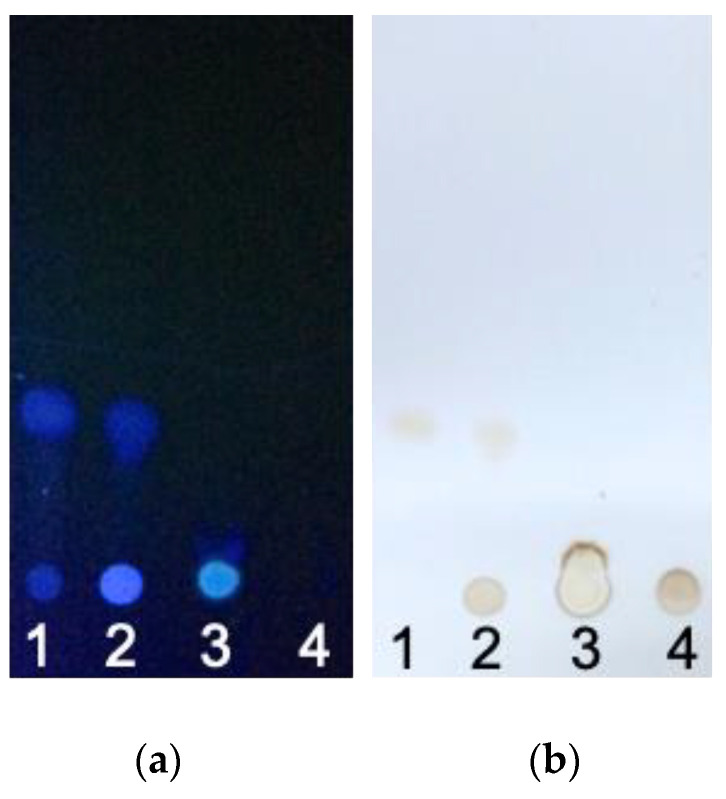
TLC of (1) resveratrol, (2) HA and resveratrol mixture (unconjugated), (3) HA-R, and (4) HA. Migration of resveratrol could be observed for free resveratrol in samples 1 and 2. HA, which did not migrate due to its molecular weight, was observed for groups 2, 3, and 4. (**a**) TLC was observed using UV light. (**b**) TLC showed hyaluronan and resveratrol after the application of heat.

Figure 1 contrasts the migration of small- and high-molecular-weight molecules. When exposed to UV light, the aromatic groups of resveratrol fluoresced blue. Free resveratrol, as shown in lanes 1 and 2, had a molecular weight of 228 g/mol and was readily carried along with the mobile phase by capillary action. However, both the fluorescence signals at the original dotted and downstream positions could be observed on lanes 1 and 2, indicating that part of the resveratrol did not migrate and was entrapped within the pores of the TLC paper. The intensity of fluorescence was higher at the dotted position for lane 2 in comparison to lane 1. This result was likely caused by decreased diffusion due to the presence of high-molecular-weight HA. When TLC paper was exposed to heat, only the migrated resveratrol signal was shown for lanes 1 and 2. In lane 2, the blot at the original dotted position was consistent with the oxidation of HA.

In contrast to resveratrol, the molecular weight of HA was approximately 1 × 10^6^ g/mole, and it could not migrate through the TLC paper. Thus, HA in lanes 2 and 4 remained in its original spotted location. For HA-R, the blot was initially circular, but it was deformed after the migration of the mobile phase. This outcome was likely due to the restriction on the movement of resveratrol that was conjugated to the backbone of HA. These results suggested that resveratrol was linked to the HA backbone. As expected, lanes 3 and 4 lacked downstream signals due either to the conjugation or lack of resveratrol, respectively. When exposed to heat, HA became oxidized and was observed in the original blotting locations, as seen in lanes 2–4.

**Figure 2:**
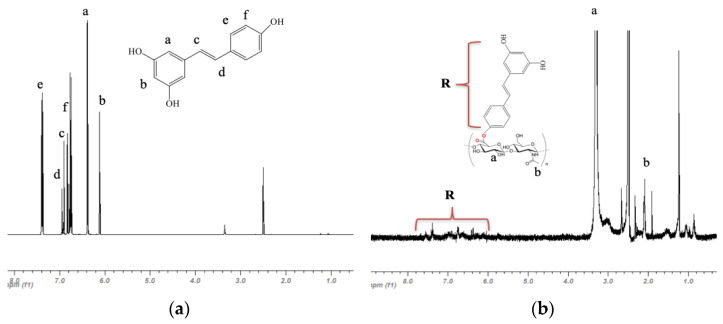
NMR analysis of (**a**) resveratrol and (**b**) HA-R. The peaks for the resveratrol group on HA-R were attenuated but present.

The nuclear magnetic resonance spectrum of resveratrol in Figure 2a indicated peaks between 6.0 and 8.0 ppm. The relative intensities and frequencies of the peaks were consistent with already published spectra [30,31,32]. This spectrum after conjugation of resveratrol to HA, though heavily attenuated, could still be identified (Figure 2b). The hydroxyl and carboxyl functional groups on HA could be observed at approximately 2.0 and 3.2 ppm, where the peaks for D-glucuronic acid (a) and N-acetylglucosamine (b) were significantly larger than resveratrol and appeared at the appropriate frequencies; they were also consistent with previously published results [23]. Although the NMR by itself could not confirm the covalent bonding of resveratrol to HA, the NMR in addition to TLC results suggested that resveratrol was successfully conjugated to HA.

**Figure 3:**
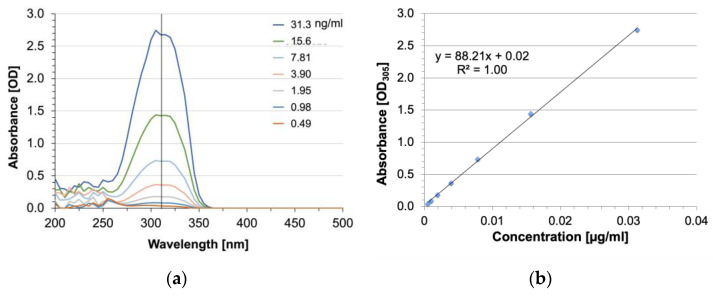
UV spectral analysis of HA-R at (**a**) various concentrations and (**b**) the standard curve. The peak wavelength was 305 nm.

UV spectral analysis revealed the absorbance of resveratrol approximately between 250 and 350 nm with a peak at 305 nm (Figure 3a). While HA-R had a distinct peak at 305 nm, HA lacked absorbance at this wavelength. An endpoint assay (Figure 3b) showed the OD signal at this wavelength decreased linearly with decreasing concentrations. A standard curve was produced using 305 nm wavelength with dissolved resveratrol (100 µM) in HA solution with 1% ethanol. Regression analysis (Table S1 in the Supplemental Data section) showed the optical density of resveratrol was related to concentrations through the equation y = (8.03 OD/µM)x + 0.11 OD with R^2^ = 0.96. Based on this standard curve, the amount of resveratrol conjugated to the backbone of HA was calculated to be 33.6% for the HA-R samples used in this study.

### 3.2. CD44 Expression

**Figure 4:**
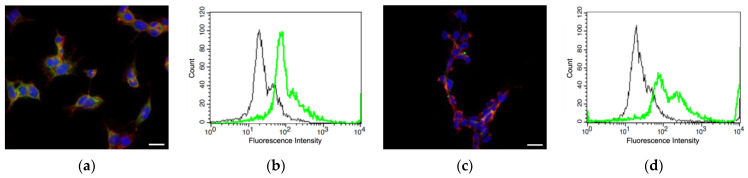

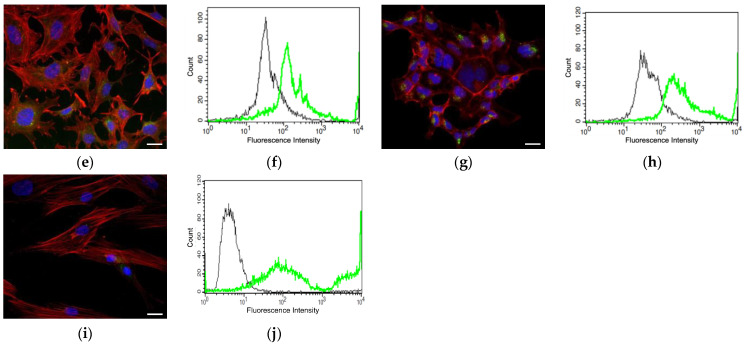
Immunofluorescence and flow cytometry assays showed positive CD44 (green), actin (red), and nuclear (blue) staining for (**a**,**b**) cortical–neuroblastoma hybrid (A1G11), (**c**,**d**) neuroblastoma (SH-SY5Y), (**e**,**f**) brain neuroglioma (H4), (**g**,**h**) human embryonic kidney (HEK293), and (**i**,**j**) fibroblast (N9). The scale bar is 10 µm. Flow cytometry data (**b**,**d**,**f**,**h**,**j**) shows positive CD 44 expression (green) and control (black).

The CD44 expression of neuroblastomas and neurogliomas was determined by immunofluorescence microscopy. The exposure settings and post-processing were identically applied for all images. All the cell lines showed positive CD44 expression by microscopy (Figure 4a,c,e,g,i). CD44 expression by confocal microcopy could be observed for A1G11, but the expression for all other cells was not as prevalent as A1G11. Although N9 showed the least CD44 staining, cells were larger, and fewer cells could be imaged within the field of view.

Due to the qualitative nature of microscopy, flow cytometry (Figure 4b,d,f,h,j) was used to characterize CD44-expressing cells. The fluorescence intensity for CD44 ranged approximately from 10^1^ to 10^3^ for A1G11, SH-SY5Y, H4 and 10^1^ to 10^4^ for HEK293 and N9, respectively. The pixel value for the histogram of A1G11 (area under the curve) was 1661, which was the highest for all the cells in this study. The pixel values for SH-SY5Y, H4, HEK293, and N9 were 1386, 1537, 1509, and 1187, respectively. Once normalized to the A1G11′s values, the resulting percentages were 100, 77, 86, 84, and 66%, respectively. These results indicated that A1G11 has the highest CD44 expression and the greatest potential for interactions with HA-R. In contrast, N9, which is a primary fibroblast, has the lowest CD44 expression and should lack interactions with HA-R. Interestingly, the CD44 expression for H4 and HEK295 were approximately equal and higher than SH-SY5Y. These results were consistent with confocal microscopy, where CD44 was clearly observed for A1G11 but barely detectable for N9.

### 3.3. HA Uptake

**Figure 5:**
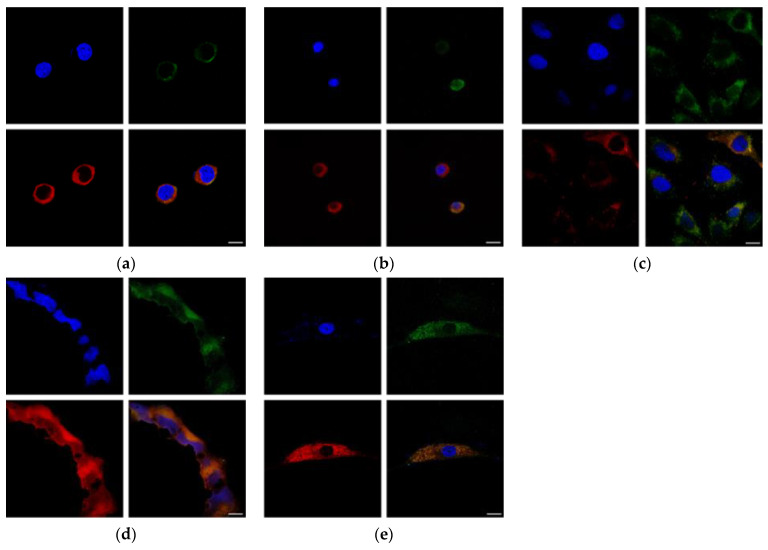
The uptake of HA-FITC (green), lysosomes (red), and nucleus (blue) (**a**) cortical–neuroblastoma hybrid (A1G11), (**b**) neuroblastoma (SH-SY5Y), (**c**) brain neuroglioma (H4), (**d**) human embryonic kidney (HEK293), and (**e**) fibroblast (N9). The scale bar represents 10 nm.

As expected, all cells showed uptake of HA. HA was observed in cells’ cytoplasm and lysosomes with varied results (Figure 5). Sectioning of the cells by confocal microscopy showed fluorescence signals in the middle of the cell instead of at the bottom or top of the cells, indicating the occurrence of uptake by either endocytosis or CD44. Consistent with other uptake studies [33,34], HA-R surrounded but did not enter the nucleus. The lysotracker studies shown in Figure 5 indicated lysosomes (stained in red) co-localized with HA-FITC (stained in green) for most cells. H4 showed the least co-localization, suggesting that the mechanisms of HA uptake could be by endocytosis/pinocytosis and by CD44. In contrast, HEK293 and N9 showed the most overlap between the CD44 and lysosomes, indicating greater reliance on endocytosis activity for HA uptake.

### 3.4. In Vitro Toxicity

**Figure 6:**
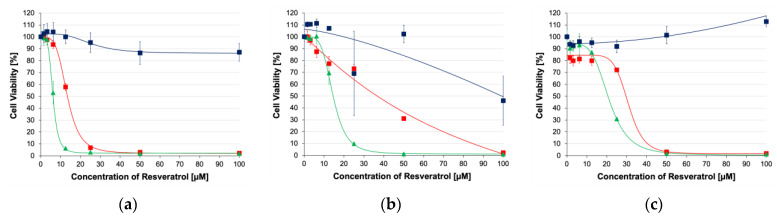

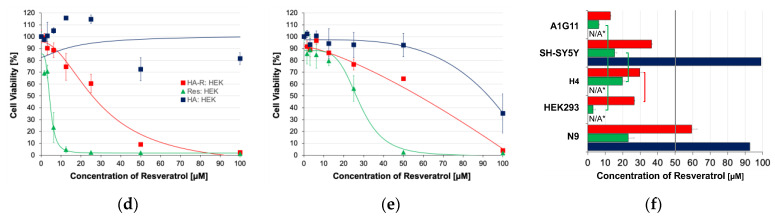
Cytotoxicity of (■) HA-R, (▲) resveratrol, and (■) HA for (**a**) cortical–neuroblastoma hybrid (A1G11), (**b**) neuroblastoma (SH-SY5Y), (**c**) brain neuroglioma (H4), (**d**) human embryonic kidney (HEK293), and (**e**) fibroblast (N9). IC_50_ values (**f**) for cells exposed to (■) HA-R, (■) resveratrol, and (■) HA. The groups connected by brackets lack statistical significance, *p* > 0.05. * Curve fits do not converge onto IC_50_ values.

The toxicities of HA-R and resveratrol are shown in Figure 6a–e for all the cell lines tested in this study. As the concentrations of HA-R and resveratrol increased, cellular survival decreased, and the dosage responses to these drugs could be modeled as symmetric sigmoidal, where the R^2^ ranged from 0.970 to 0.999 (Table S1 in the Supplemental Data also has curve fits for resveratrol and HA). In addition, randomized phase-contrast images for cells exposed to HA-R and resveratrol with 1% ethanol are given in the supplementary data (Figure S1).

Resveratrol derivatization to HA generally attenuated the toxicity for all the cells in this study in comparison to the same drug dissolved in a 1% ethanol solution. Without the addition of ethanol, the drug cannot be dissolved in an aqueous medium such as a tissue culture medium and lacks efficacy. In contrast, HA-R results did not use any ethanol. The reductions in toxicity were greater with breast cancers that were previously reported with HA-R, where HA conjugation generally enhanced efficacies [22,23]. The differences could be due to the levels and/or isoforms of CD44 and expression of other HA receptors. For example, Shah et al. reported that the percentages of positive cells binding to HA for three breast cancer cell lines (MDA-MB-231, MDA-MB-157, and MCF7) in culture were almost 100%, but the strength of attachment to HA receptors seemed to be significantly weaker for MCF7, as the amount of binding decreased with the increasing competition of non-labeled HA [23]. CD44s were also dominantly expressed by MDA-MB-231, an aggressive strain, and accounted for approximately 60% of expression [35]. The balance of the CD44 population was the variant isoforms containing CD44v2–CD44v10. In contrast, CD44v8 was the dominant isoform for MCF7, which was a nonaggressive strain, and CD44s accounted for as little as 8% of total CD44 expression [35]. In contrast to the breast cancer cells, the percentage of positive SH-SY5Y cells binding to HA ranged from 10 to 45% [36,37]. Since breast cancer cells were likely to express higher levels of HA receptors than neuroblastomas, the attachment and uptake of HA-R were likely to be lower.

The IC_50_ (Figure 6f) values for neuroblastoma (A1G11 and SH-SY5Y) and neuroglioma (H4) cell lines tested in this study increased by 2.1, 2.4, and 1.5 times, respectively, when exposed to HA-R in comparison to resveratrol. Therefore, the toxicity results were well correlated with CD44 expression, where HA-R had the highest toxicity to A1G11 (IC_50_ = 13.4 µM) and CD44 expression relative to other cells in this study. HA-R’s toxicity in comparison to A1G11 was significantly lower for SH-SY5Y (IC_50_ = 38.5 µM, *p* = 0.001), H4 (IC_50_ = 29.7 µM, *p* = 0.001), HEK293 (IC_50_ = 26.5 µM, *p* = 0.001), and N9 (IC_50_ = 59.4 µM, *p* = 0.001) and correlated very well with CD44 expression (100%, 77%, 86%, 84%, and 66%, respectively). A systemic comparison of the *p*-values is given in Table S2 (see the Supplemental Data section). When compared to previously published breast cancer cell lines, the IC_50_ values were lower for MDA-MB-231 (IC_50_ = 5.5 µM) but higher for MCF7 (IC_50_ = 65.5 µM) [22,23]. These outcomes for MDA-MB-231 compared to neuroblastomas and neuroglioma could be due to the amounts of overall CD44 expression that resulted in higher HA attachment and uptake. However, the attachment for MCF7 was higher in comparison to neuroblastomas and neuroglioma, but the higher IC_50_ could be due to isoform expression. Relatively low or even lacking CD44 expression for neuroblastomas typically indicates a better prognosis for patients with both the standard isoform and splice variants [38,39].

Interestingly, resveratrol’s toxicity was the greatest for HEK and A1G11 (IC_50_ = 6.2 and 4.1 µM, respectively). For H4, SH-SY5Y, and fibroblasts, the IC_50_ values were 20.6, 15.5, and 26.3 µM, respectively. Although these values were within therapeutic concentrations, 1% ethanol was used to aid in the solubility of resveratrol. Since the adjuvants used in clinical trials lack the supplementation of ethanol or other organic solvents, the therapeutic benefits of resveratrol have been severely limited, and many clinical trials with resveratrol have failed, as previously reviewed [40]. In contrast, HA-R could be dissolved only using aqueous solutions due to the ability of HA to form extensive hydrogen bonding with water. The IC_50_ values when exposed to HA were greater than 90 µM or could not be obtained due to cell growth in response to increasing HA concentrations. As expected, HA by itself was generally non-cytotoxic unless it was at high concentrations, and only SH-SY5Y and N9 exhibited a very low degree of toxicity, where IC_50_ ≥ 92.7µM or could not be calculated due to cell growth increases (then being labeled N/A in Figure 6f). Finally, hyaluronan conjugate with a fluorescent molecule can easily be solubilized in an aqueous medium, and preliminary studies show it can be injected into the tail vein of mice. After 6 h, the hyaluronan conjugate is dissipated from the tail veil (Figure S2 in the Supplemental Data section).

## 4. Conclusions

Chemotherapeutics should have enough toxicity to kill tumors while minimizing unwanted side effects. Tissues composed of terminally differentiated cells, such as nervous system tissues, have limited capacity to regenerate and/or require a prolonged time to recover from injury. The application of targeted therapy to these tissues is especially paramount in preventing damage to healthy cells. Targeting a uniquely expressed receptor is highly desirable but not possible for most diseased tissues since they originated from normal cells. Developing targeted therapy that recognizes overexpressed receptors such as CD44 could be a valuable strategy for improving the effectiveness of existing chemotherapeutics and minimizing collateral damage to healthy cells. For normal brain tissues, CD44 was rarely observed in the cortex but was more predominant in the white matter [41,42,43]. Adult neurons either expressed CD44 at very low levels or were CD44-negative, but this receptor was upregulated during injury and/or the onset of inflammation [41,43,44,45,46,47,48]. This differential expression could be highly advantageous for treating a variety of brain tumors. Since the cortical–neuroblastoma cells (A1G11) responded well to HA-R, even when the derivatization attenuated the efficacy as compared to resveratrol in 1% ethanol solution, HA-R should be explored further as a potential receptor-mediated therapeutic for tumors with similar levels of CD44 expression. Treatment for SH-SY5Y and H4 and similar types of tumors, in contrast, will likely require either targeting a different receptor or conjugating chemotherapeutics with higher toxicity. Therefore, actively targeting overexpressed receptors could be a viable strategy for developing new therapeutics for brain tumors.

## Data Availability

Raw data will be made available upon request to the corresponding author.

## Supplementary Materials

The following supporting information can be downloaded at: https://www.mdpi.com/article/10.3390/pharmaceutics16060836/s1, Figure S1: Comparison of phase-contrast images of A1G11, SH-SY5Y, H4, HEK 293, and N9 cells exposed to HA-R and resveratrol with 1% ethanol; Figure S2: Tail vein injection of HA-rhodamine after (a) 1, (b) 3, and (c) 6 h; Table S1: Curve fit for toxicity studies; Table S2: ANOVA with Tukey’s post hoc test for IC_50_ (*p*-values).
