# Supplementary material for: Derivatization of Hyaluronan to Target Neuroblastoma and Neuroglioma Expressing CD44
Source: Pharmaceutics. 2024 Jun 20;16(6):836. doi: 10.3390/pharmaceutics16060836 (PMC11207210; doi:10.3390/pharmaceutics16060836)
Supplement: Supplementary file 1 [file pharmaceutics-16-00836-s001.zip › pharmaceutics-3008344-supplementary.pdf]

Table S1 – Curve Fit for the Toxicity Studies

		Curve Fit Equation	R^2
HA-R	A1G11	$2.0 + [(99.3 – 2.0) / (1 + (X/13.3)^{4.3})]$	0.999
	SH-SY5Y	$-92.2 + [(99.9 + 92.2) / (1 + (X/95.0)^{1.1})]$	0.980
	H4	$1.7 + [(84.7 – 1.7) / (1 + (X/30.9)^{8.1})]$	0.970
	HEK293	$-10.3 + [(95.7 + 10.3) / (1 + (X/29.6)^{2.0})]$	0.976
	N9	$-5.8x10^{7} + [(91.8 + 5.8x10^{7}) / (1 + (X/2.5x10^{6})^{1.3})]$	0.966
Res	A1G11	$2.2 + [(100.0 – 2.2) / (1 + (X/6.1)^{4.6})]$	0.999
	SH-SY5Y	$1.2 + [(100.0 – 1.2) / (1 + (X/14.5)^{4.4})]$	0.983
	H4	$0.5 + [(94.0 – 0.5) / (1 + (X/21.2)^{4.4})]$	0.996
	HEK293	$1.8 + [(85.3 – 1.8) / (1 + (X/4.5)^{3.5})]$	0.956
	N9	$-0.6 + [(88.3 + 0.6) / (1 + (X/28.0)^{4.6})]$	0.980
HA	A1G11	$86.2 + [(102.6 – 86.2) / (1 + (X/25.4)^{3.6})]$	0.955
	SH-SY5Y	$-6.4x10^{7} + [(106.2 + 6.4x10^{7}) / (1 + (X/2.8x10^{6})^{1.4})]$	0.679
	H4	$1.8x10^{7} + [(94.3 – 1.8x10^{7}) / (1 + (X/1.3x10^{5})^{1.9})]$	0.873
	HEK293	$82.6 + [(101.1 – 82.6) / (1 + (X/16.5)^{-1.4})]$	0.976
	N9	$-1.0x10^{7} + [(97.4 + 1.0x10^{7}) / (1 + (X/2.9x10^{3})^{3.6})]$	0.975

Table S2 – ANOVA with Tukey post hoc test for the IC_{50} (p-values)

		HA-R				
		A1G11	SH-SY5Y	H4	HEK293	N9
HA-R	A1G11	---	0.001	0.001	0.001	0.001
	SH-SY5Y	---	---	0.032	0.001	0.001
	H4	---	---	---	0.728	0.001
	HEK293	---	---	---	---	0.001
	Fib	---	---	---	---	---

		Res				
		A1G11	SH-SY5Y	H4	HEK293	N9
HA-R	A1G11	0.038	0.900	0.025	0.001	0.001
	SH-SY5Y	0.001	0.001	0.001	0.001	0.001
	H4	0.001	0.001	0.001	0.001	0.039
	HEK293	0.001	0.001	0.032	0.001	0.649
	N9	0.001	0.001	0.001	0.001	0.001

		Res				
		A1G11	SH-SY5Y	H4	HEK293	N9
Res	A1G11		0.002	0.001	0.704	0.001
	SH-SY5Y		---	0.325	0.001	0.009
	H4		---	---	0.001	0.678
	HEK293		---	---	---	0.001
	N9		---	---	---	---

Figure S1 – Comparison of the phase contrast images of A1G11, SH-SY5Y, H4, HEK 293, and N9 cells exposed to HA-R and resveratrol with 1% ethanol

A1G11 | **HA-R** | **Res. with Ethanol**

Concentration [μM]

100

50

25

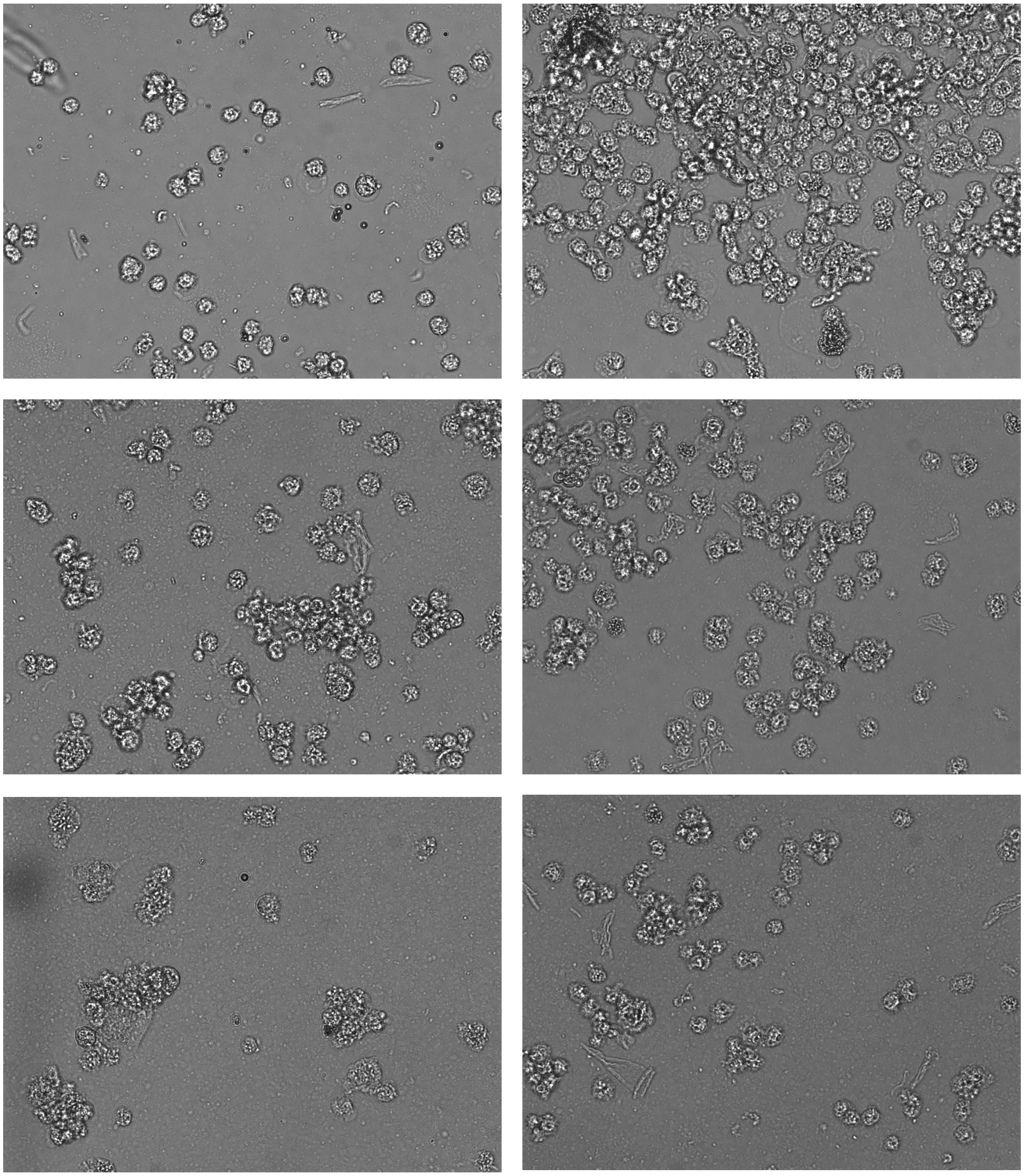

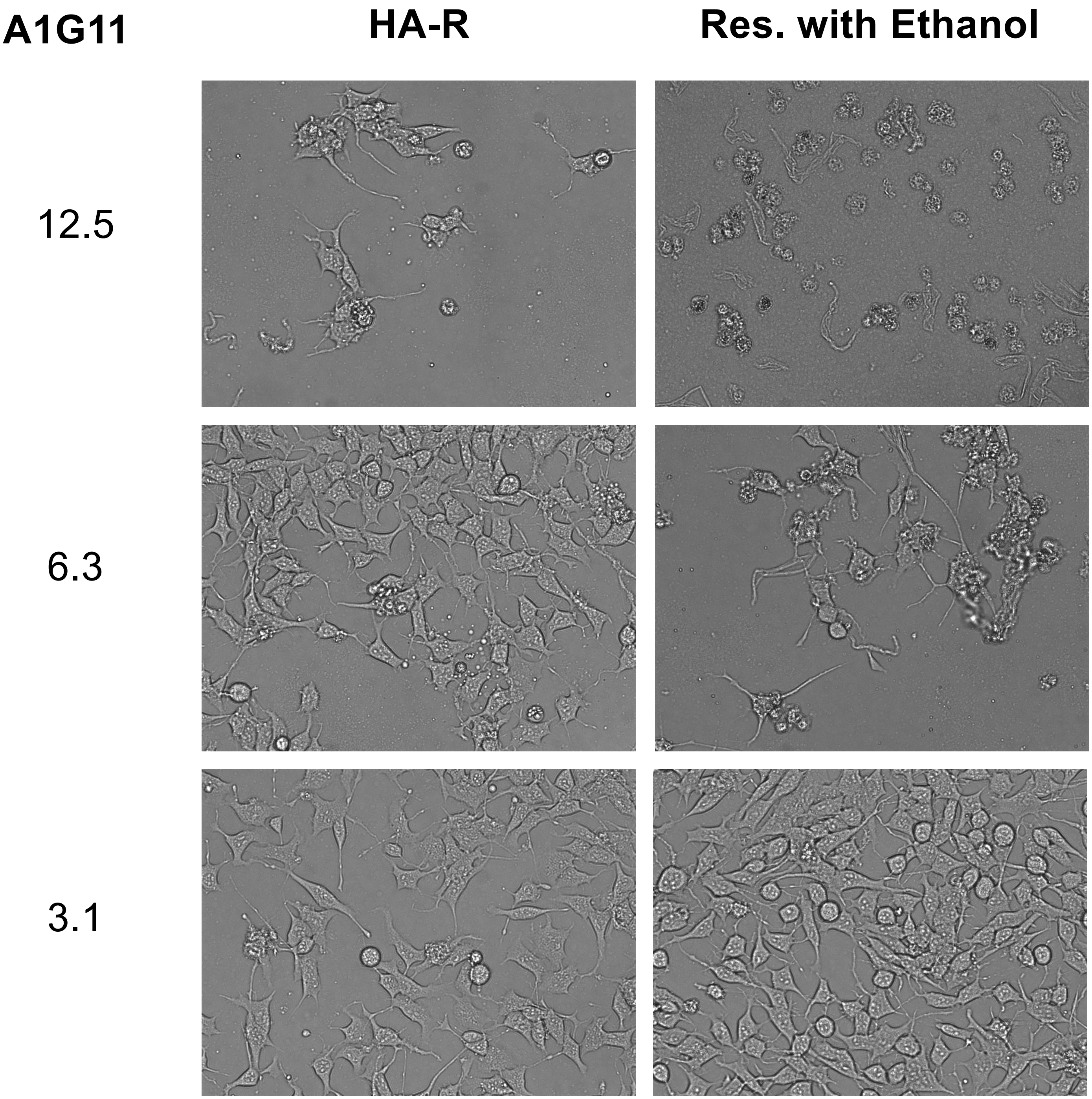
A1G11
HA-R
Res. with Ethanol
12.5
6.3
3.1

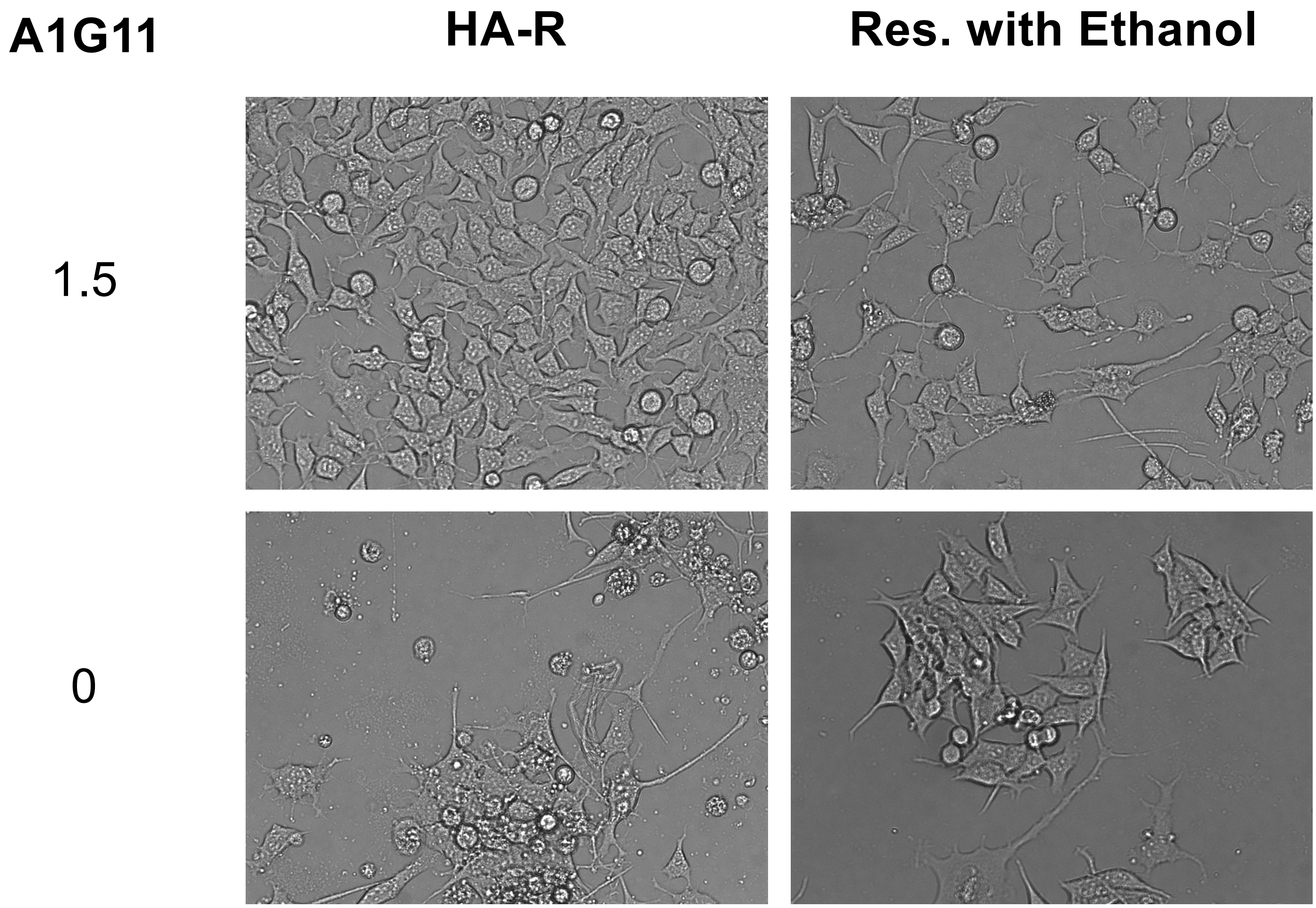
A1G11
HA-R
Res. with Ethanol
1.5
0

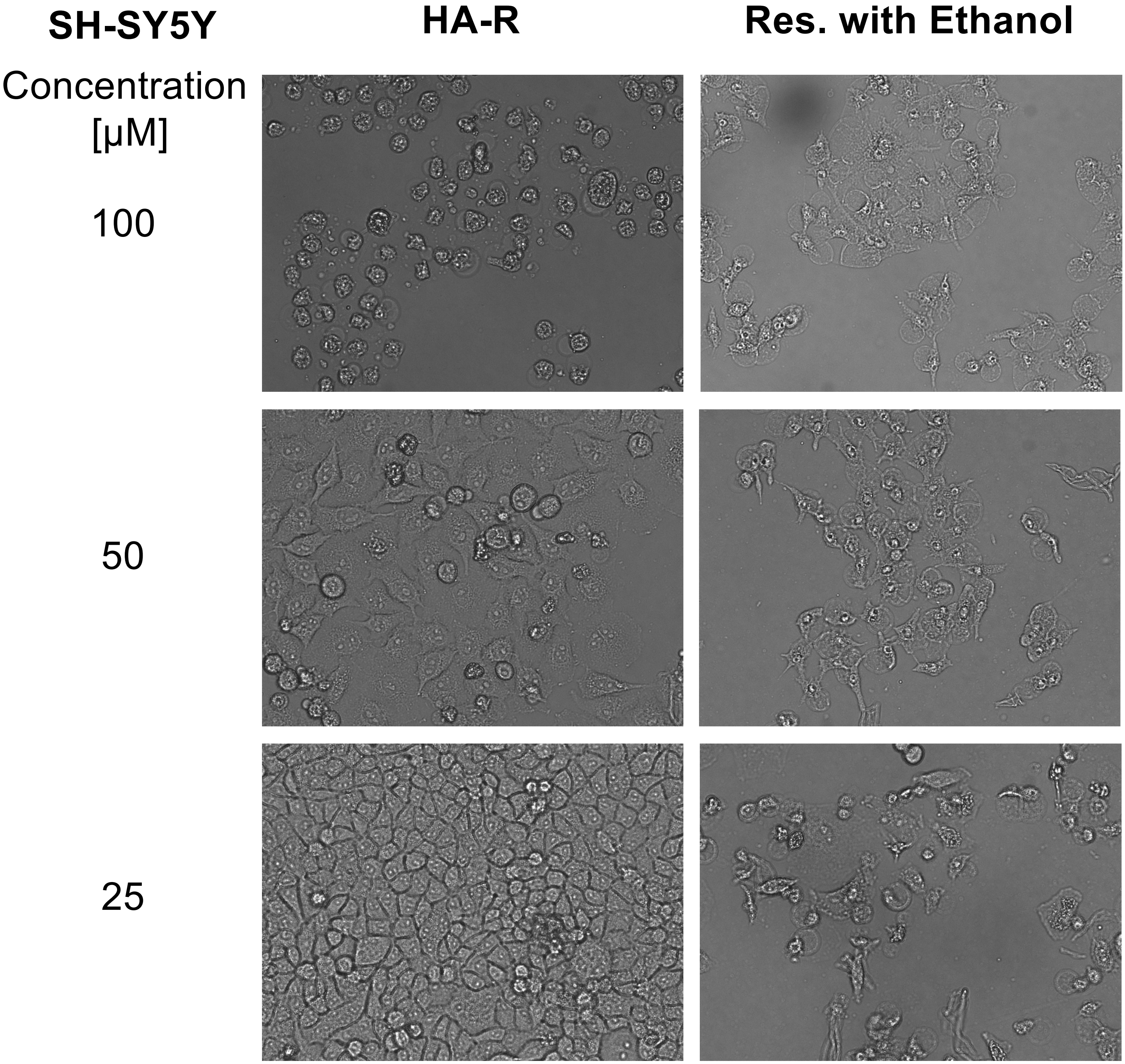
SH-SY5Y
HA-R
Res. with Ethanol
Concentration [μM]
100
50
25

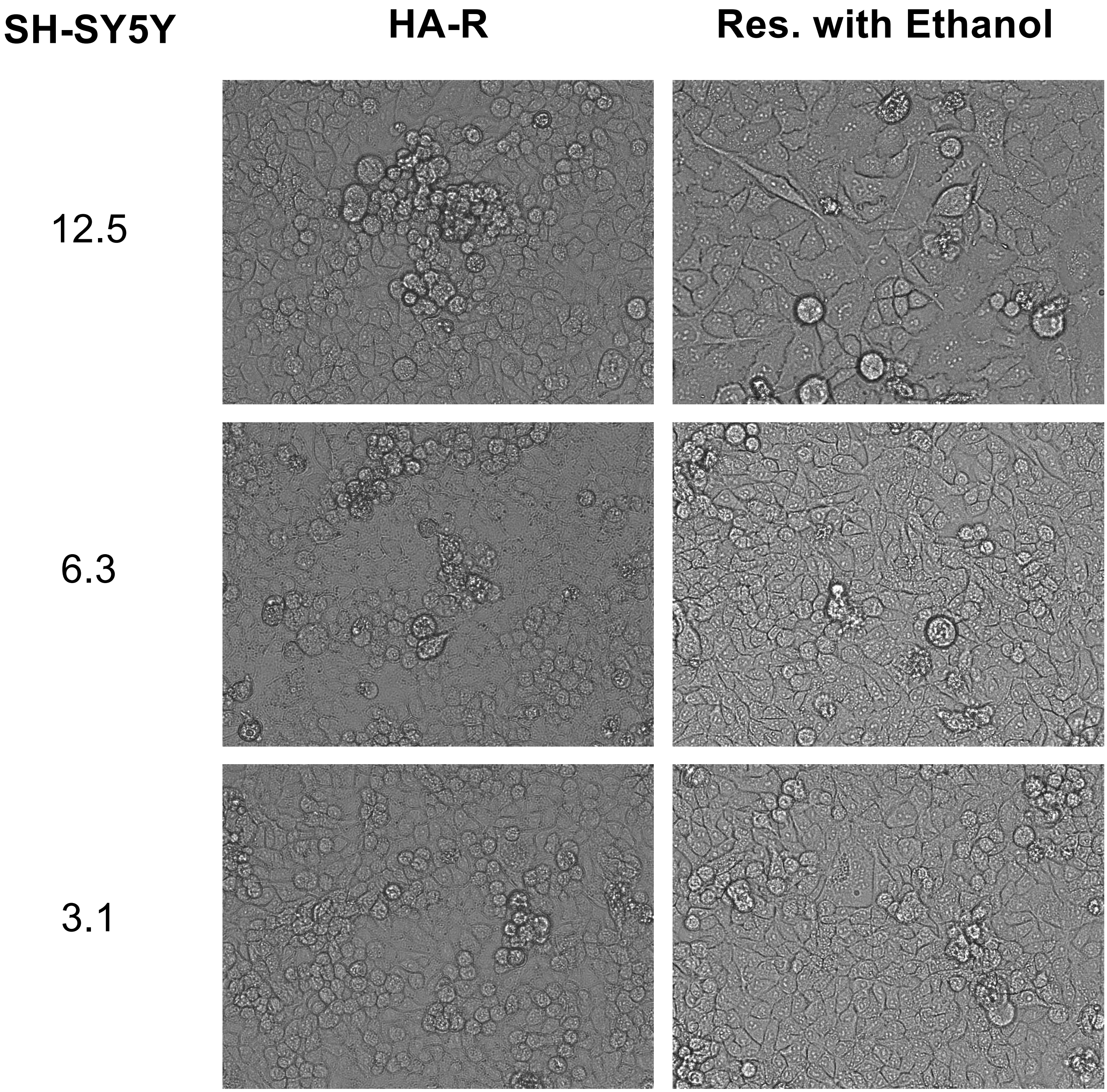
SH-SY5Y
HA-R
Res. with Ethanol
12.5
6.3
3.1

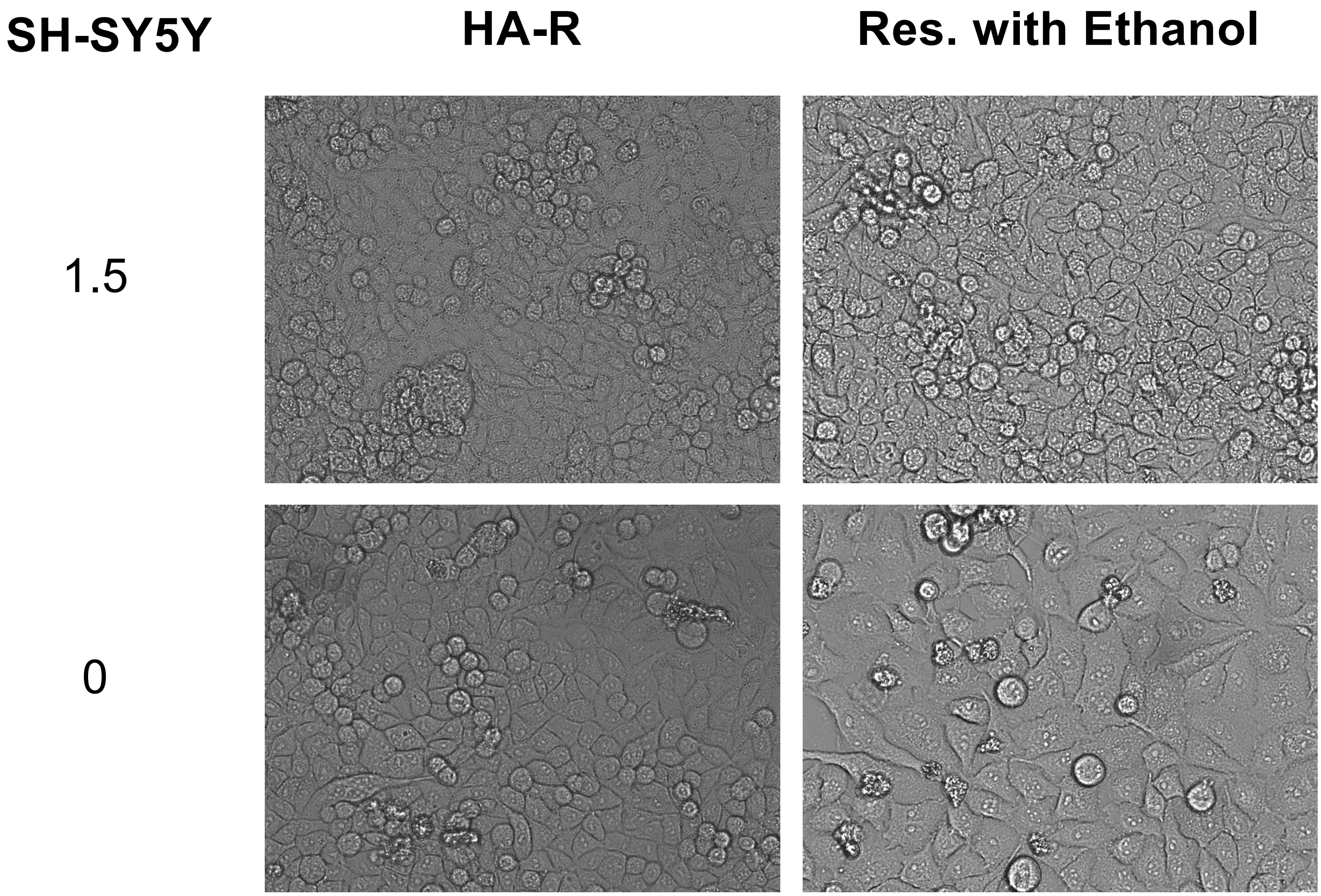
SH-SY5Y
HA-R
Res. with Ethanol
1.5
0

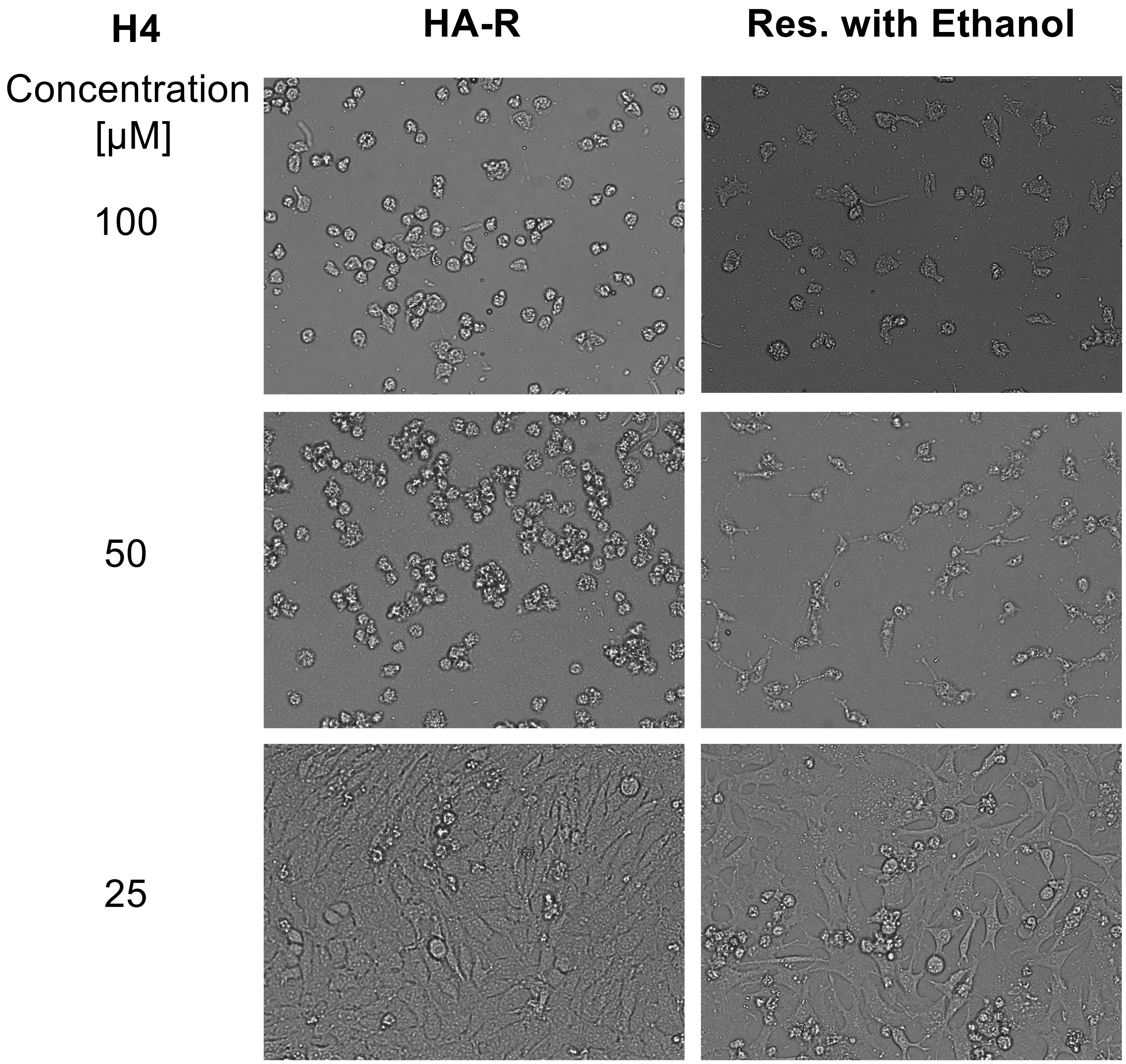
H4
HA-R
Res. with Ethanol
Concentration [μM]
100
50
25

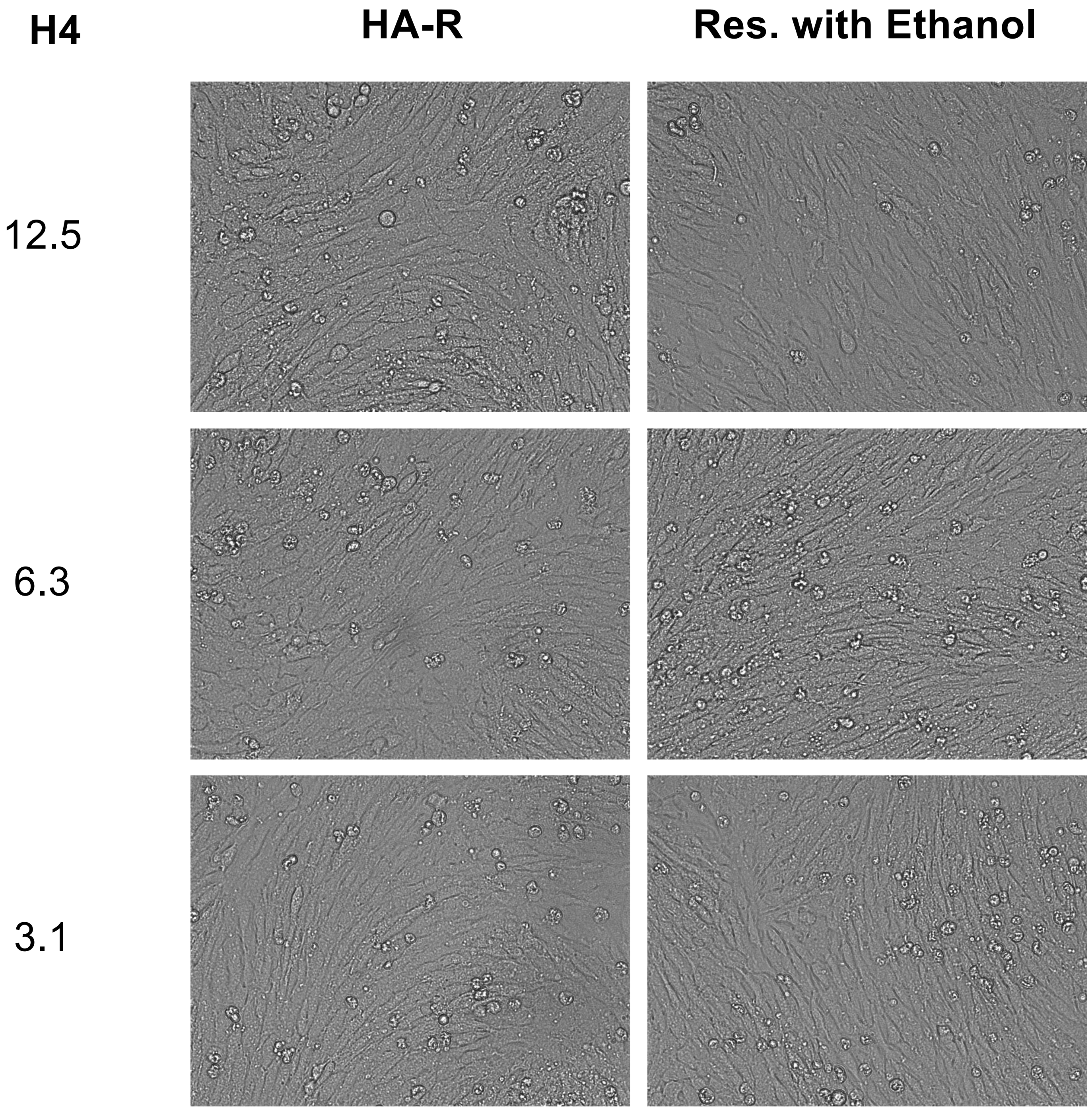
H4
HA-R
Res. with Ethanol
12.5
6.3
3.1

H4 **HA-R** **Res. with Ethanol**

1.5

0

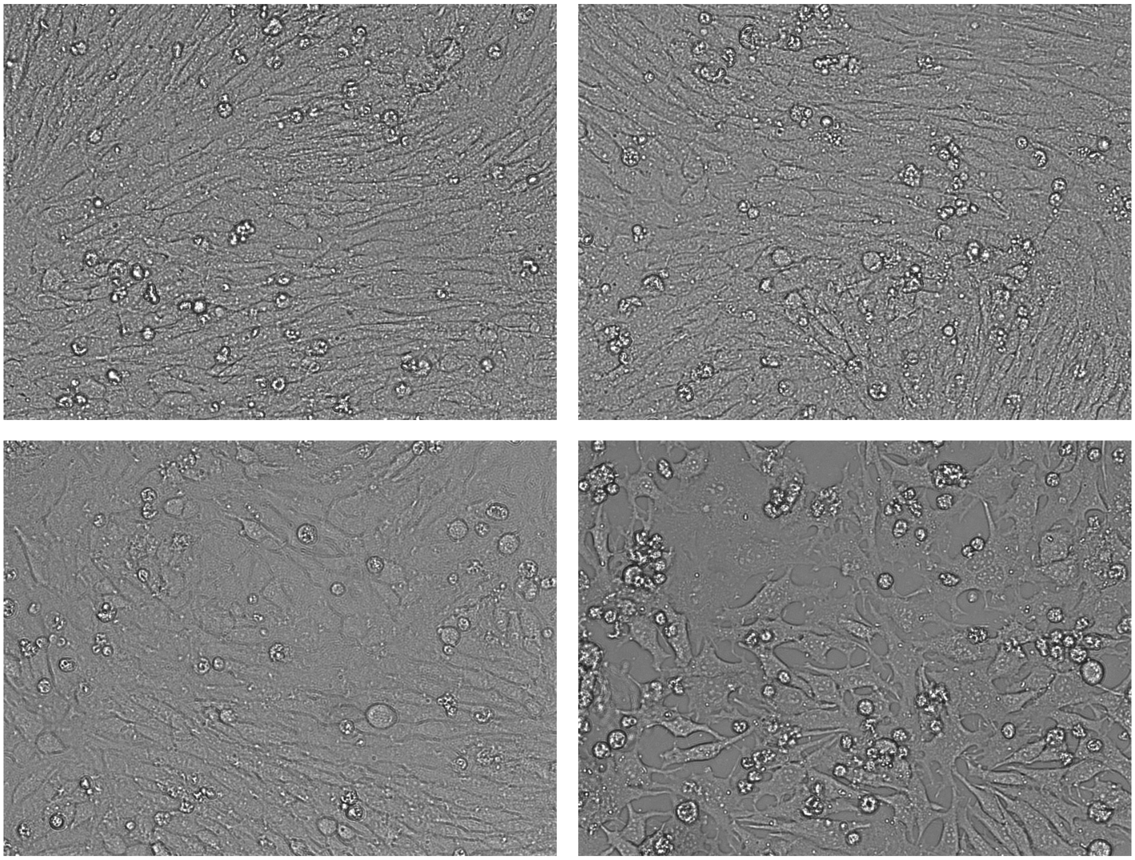

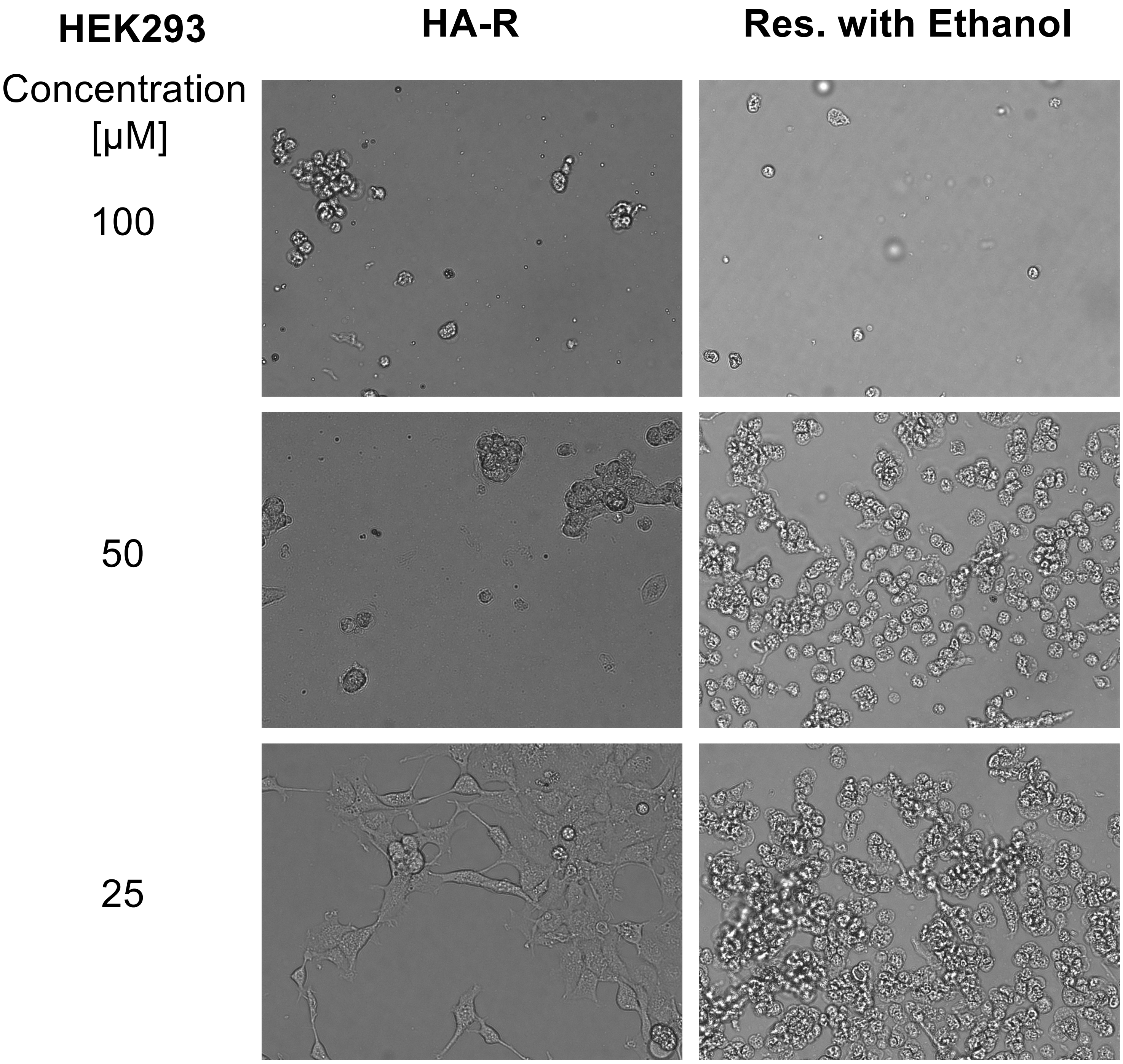
HEK293
HA-R
Res. with Ethanol
Concentration [μM]
100
50
25

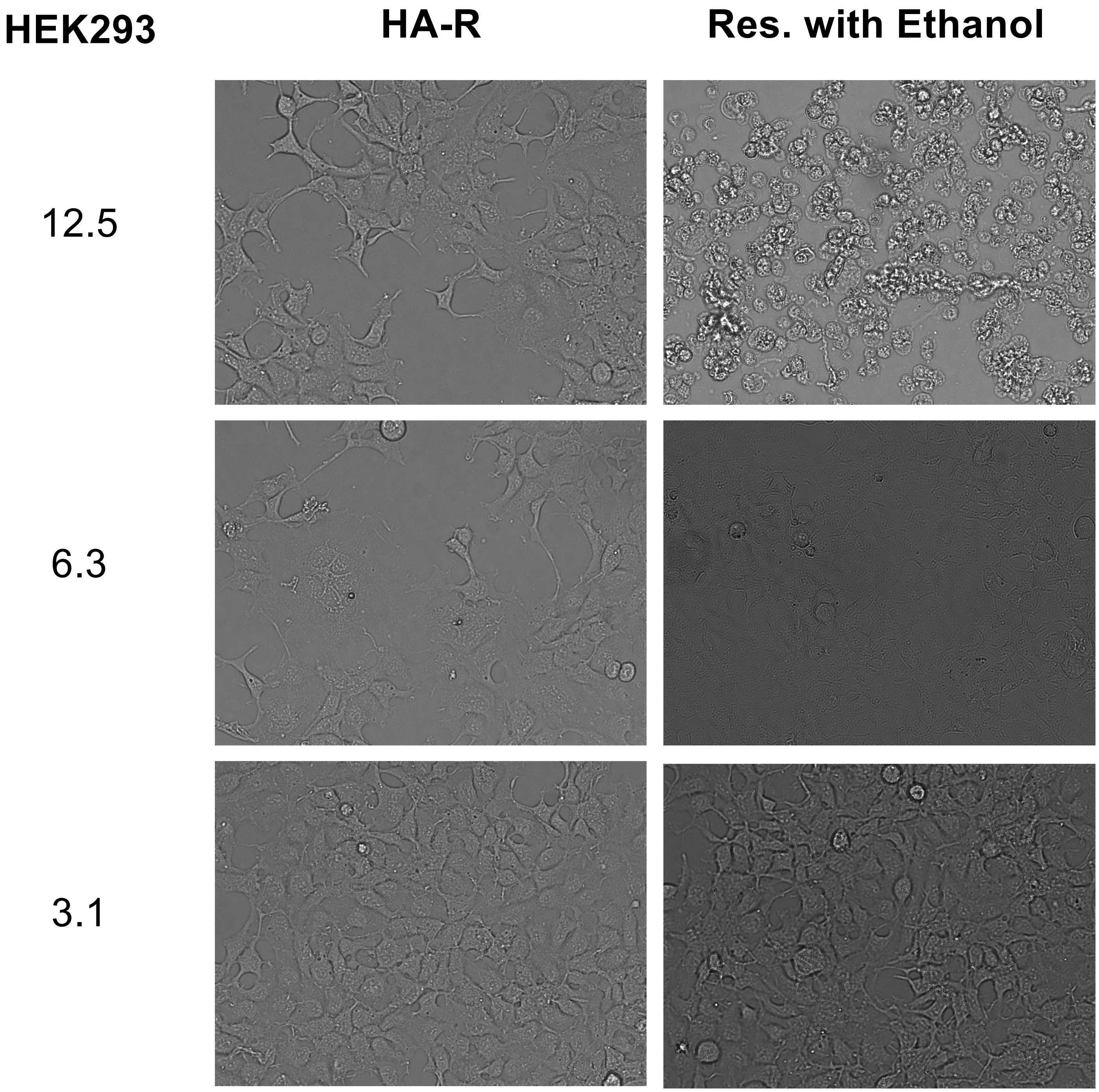
HEK293
HA-R
Res. with Ethanol
12.5
6.3
3.1

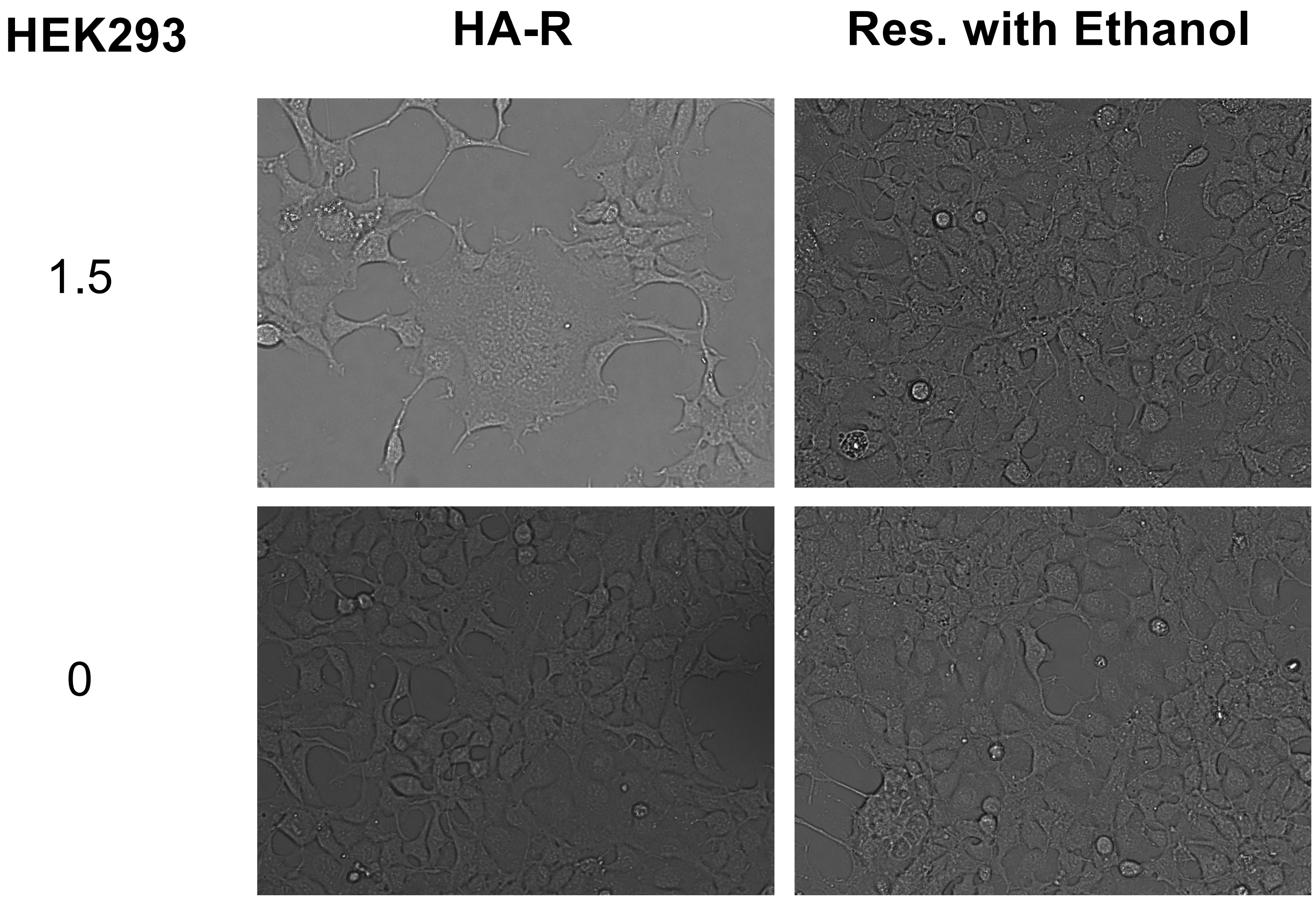
HEK293
HA-R
Res. with Ethanol
1.5
0

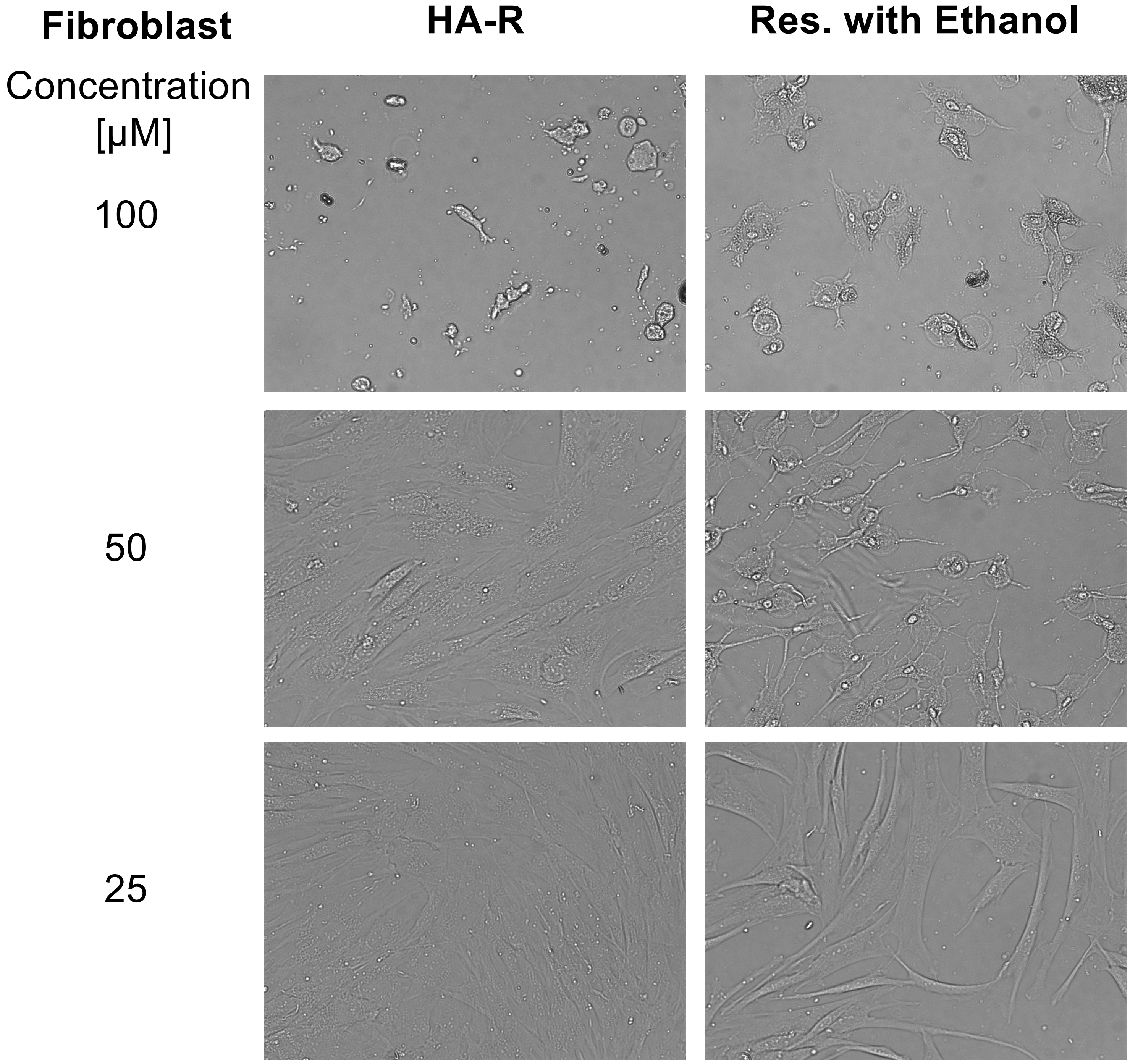
Fibroblast
HA-R
Res. with Ethanol
Concentration
[μM]
100
50
25

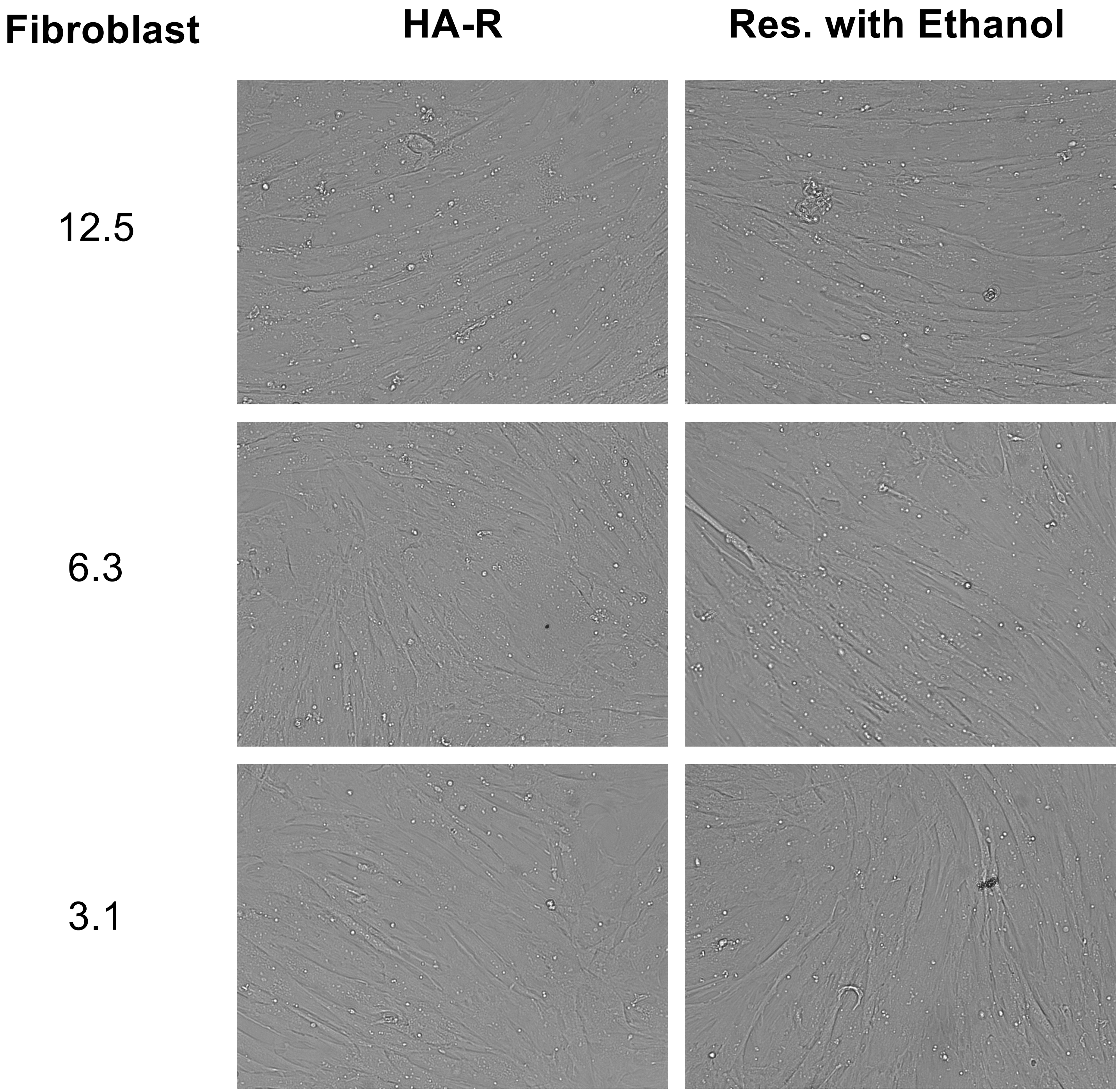
Fibroblast
HA-R
Res. with Ethanol
12.5
6.3
3.1

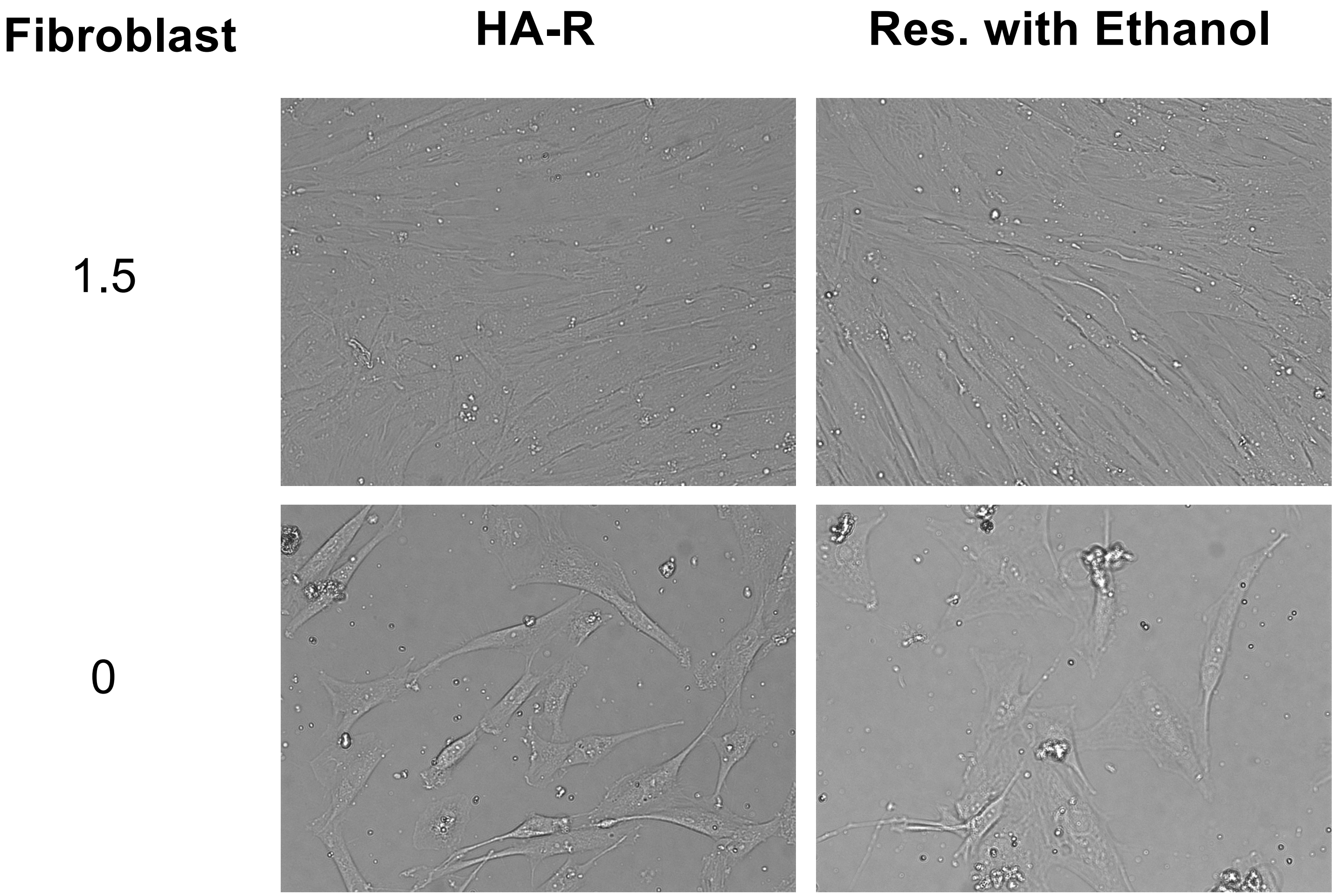
Fibroblast
HA-R
Res. with Ethanol
1.5
0

Figure S2 – Tail vein injection of HA-rhodamine after (a) 1, (b) 3, and (c) 6 hours

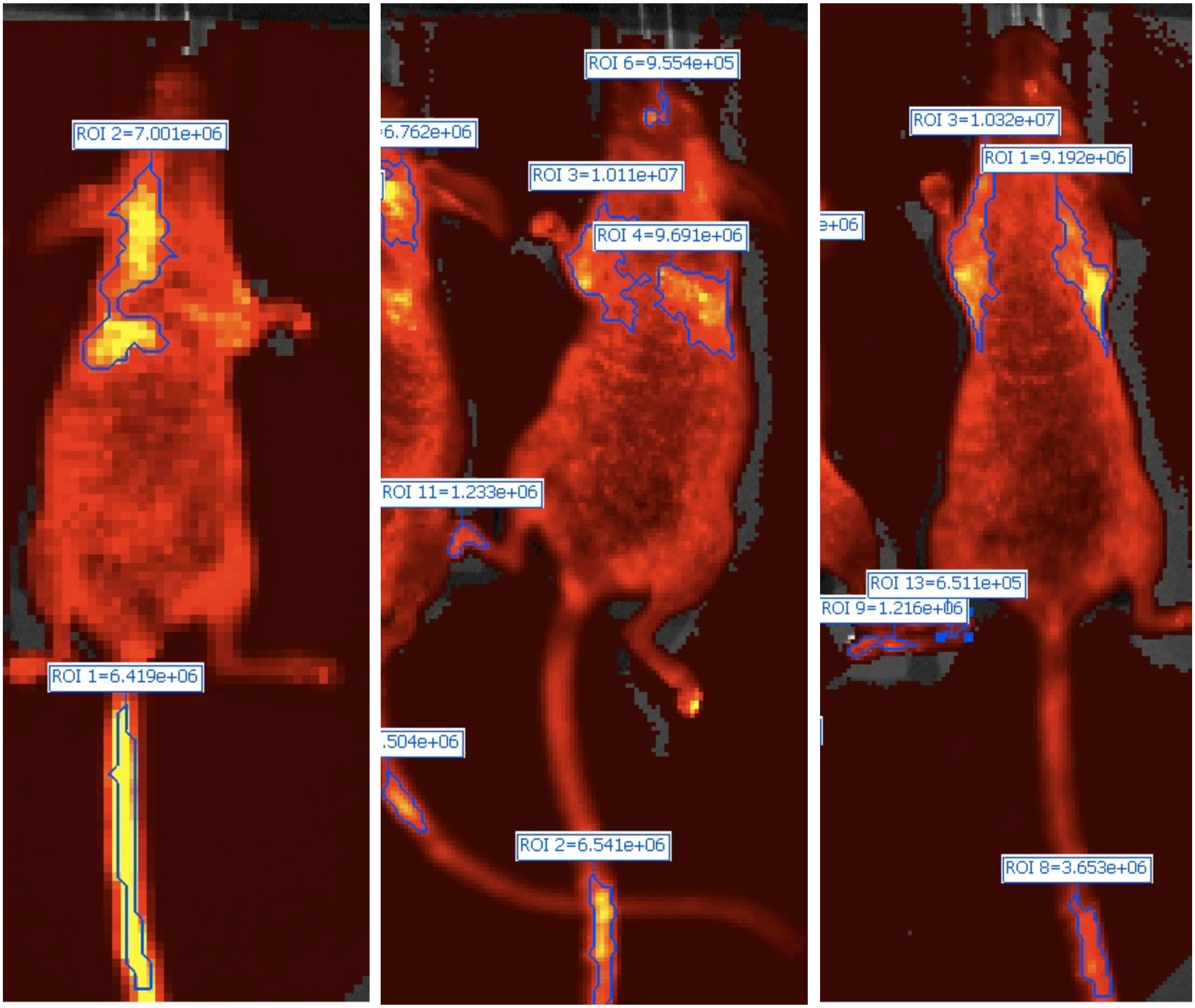

(a) (b) (c)
